# Affective polarization in a word: Open-ended and self-coded evaluations of partisan affect

**DOI:** 10.1371/journal.pone.0310772

**Published:** 2025-01-16

**Authors:** Spencer Kiesel, Sharif Amlani

**Affiliations:** Department of Political Science, University of California, Davis, Davis, California, United States of America; University of Hamburg: Universitat Hamburg, GERMANY

## Abstract

The literature finds that partisanship drives negative emotional evaluations of out-partisans. Yet, scholars base these insights on measures–like thermometers, candidate evaluations, and social-distance measures–that discount the sentiment attached to individuals’ negative attitudes. We introduce a unique measure of affect capturing the motivation underpinning partisans’ attitudes. Our measure asks respondents for one-word to describe voters in their party and the opposing party. Then respondents code the sentiment behind their word choice themselves. Together, our measure produces qualitative and quantitative measures of respondents’ affect. We find that our self-coded open-ended measure has strong face validity and correlates strongly with existing affect measures. This measure advances our understating of partisan affect by allowing scholars a window into respondents’ state of mind. Scholars can easily apply our measure’s procedure beyond partisanship to other groups of interest.

## Introduction

Partisanship, as a social identity, has led to increasingly negative evaluations of out-party members, also known as affective polarization [1–3]. But, to what extent are these evaluations negative and what is the sentiment behind them?

To measure the dislike between partisans, scholars rely on thermometer measures, candidate evaluations, and social-distance measures of partisans’ willingness to engage with opponents [2, 4]. However, these measures suffer from a common weakness: closed answered scales [5]. These measures use a numerical scale to quantify respondents’ affect. While this approach makes quantitative analysis easier, it does not capture the motivations underlying their feelings, making it difficult to understand the mechanisms driving respondents’ affective evaluations.

Therefore, scholars are cross-pressured: how can we measure *what* people feel and *why* they feel it? We argue that existing measures provide a decent evaluation of respondents’ affect but do not illuminate the motivations underlying partisans’ evaluations. In this article, we present a new protocol to measure affect. Our two-question measure starts by asking respondents to report one word characterizing partisans who share their party identification and one word characterizing partisans with the opposing party identification. Then, we ask respondents to code the sentiment behind their own word on a seven-point scale from extremely negative to extremely positive, with neutral as the midpoint option. This procedure produces a word and a numeric score both generated by the respondent. The word provides insight into what motivates a respondent’s affect, and the score tells us the intensity of that affect. In combination, scholars obtain a qualitative and a quantitative evaluation of the respondents’ affect towards in and out partisans.

Our measure is grounded in Zaller’s Receive-Accept-Sample model of public opinion formation [6]. When prompted, respondents draw from the distribution of their top-of-mind characterizations of partisans and provide a word that matches the underlying sentiments they hold. Thus, the words reflect each respondent’s most salient top of mind considerations. When we evaluate the robustness of our measurement procedure, we find that our one-word measure has high internal and external validity. First, when we look at respondents’ word selection and the code they assign to their word, the measure performs as we expect: respondents have positive evaluations of in-partisans and negative evaluations of out-partisans. Second, when we compare our measure of affect with established measures of affect, we find the measures are strongly related but not perfectly related, suggesting that our measure contributes a unique perspective to our understanding of the latent dimensions of affect.

Our two-question measure provides reliable and valid estimates of respondents’ affect and has at least four major benefits. First, open-ended responses give researchers a window into the respondents’ state of mind that closed ended responses do not. Second, our measure more directly captures respondents’ affect by eliciting spontaneous evaluations open to whatever natural language occurs to respondents. Third, we overcome barriers associated with open-ended responses by asking respondents to score their own words. This procedure quantified open ended responses underlying affective evaluations without the difficulties associated with matching open-ended responses to sentiment dictionaries. Finally, we show that our measure is comparable to existing measures but is not perfectly correlated, suggesting that our measure captures a unique affect of the latent concept of affect. Our measure balances the competing concern researchers face between easy to use closed ended items and the rich but unwieldy data provided by open ended questions.

Furthermore, our measure produces findings beyond what purely quantitative measures can show that underscore the degree to which American politics occurs in a climate where partisans not only disagree with opponents on policy issues but view them as illegitimate or malevolent. The words respondents use to describe out-partisans—such as “sheep,” “sheeple,” “misguide,” “uninformed,” “misinform,” “uneducated,” “brainwash”—reflect this feature of contemporary politics. These demeaning and patronizing characterizations highlight the abyss of affective polarization between both parties in a much more visceral way than thermometer averages. They reveal a deeply entrenched in-group versus out-group mentality, signal distrust and dehumanization of opponents, and reduce complex policy disagreements to the impulsive vilification of the opposing side. These words highlight that some Americans not only see policy differences between partisans but think in harsh pejorative terms.

In the following five sections, we discuss how our measure relates to existing methods; present the data and methods we used to construct and test our measure; provide evidence of internal validity; provide evidence of external validity by comparing our estimates to those produced by well established measures such as thermometers; and argue that the qualitative data produced by one-word measures can provide novel insights about the dimensions of affect. Finaly, we conclude by summarizing by summarizing the limitations of our measure and outlining future work.

## Measuring affect

Affect refers to a feeling or emotion [7]. When applied to a group in survey research, affect refers to the feelings or emotions a respondent has towards that group [8–10]. Scholars studying partisan affect draw on three measures of affect: thermometers, candidate evaluations, and social-distance measures. The most common tool scholars use to measure partisan affect are feeling thermometers [1, 2, 11]. Respondents rate in and out-party on a scale ranging from 0 to 100, as a representation of their feelings toward each group [3, 12]. A second measure for partisan affect is respondents’ evaluations of major party leaders [13–15]. In the United States, respondents evaluate the Republican and Democratic candidates for president on 10-point scales of overall favorableness, trustworthiness, recklessness, and to what extent the respondent shares their values [13]. Abroad, Reiljan (2020) [14] developed the Affective Polarization Index (API). This index takes survey responses using 0 to 10 like–dislike scale for each party [14] and their leader [15] and applies a weighted average of the difference between in-party and out-party like-dislike scores. The index weights this difference by the electoral size of each party. A third measure of affect is social-distance measures that use social distance questions, such as those introduced by Iyengar, Sood, and Lelkes (2012) [16] that ask respondents how troubled they would be by a family member marrying an out-party member.

While scholars use these measures of affect widely and we fully acknowledge their usefulness, they are not without their limitations. First, survey researchers did not develop thermometer scales to measure affect [12]. As Wilcox, Sigelman, and Cook (1989, 251) [17] warn, “if one uses a feeling thermometer to measure affect toward any particular group, one will have to bear in mind that some respondents respond to feeling thermometers in an unusual manner. This may pose a particular problem when feeling thermometers are used to identify supporters of particular social groups.” Feeling thermometers also suffer from “inter-personal incomparability” [18, 19]. That is, people tend to interpret feeling thermometer scales differently making comparing evaluations across individuals tenuous [18, 19]. Additionally, respondents tend to bias their responses towards the ends of both scales and around the 50 mark, suggesting that respondents do not use the full range of the scale and some limit themselves to certain areas of it [17]. Weisberg and Miller (1980) [12] find that mislabeling thermometer scores may over or under estimate respondents’ actual feeling.

Second, party leader ratings exclusively focus on elites as potential drivers of affect, which may or may not be the actual reason behind a respondent’s dislike for the out-party. Furthermore, the use of like-dislike scales or trait rating scales means that researchers select the dimensions that respondents rate leaders on. This method imposes the researchers’ chosen dimensions onto the respondents, who are then limited to providing information only within the predefined parameters set by the researchers, rather than based on their own perspectives. Third, social distance measures like those used by Iyengar, Sood, and Lelkes (2012) [16] capture respondents’ willingness to *engage* with out-partisans not their *feelings* about them, which ultimately defines affect. Social-distance measures are a consequence of negative affect toward opposing partisans and do not capture their state of mind *about* them.

In addition to their individual weaknesses, existing measures compress affective evaluations into closed-answer scales, preventing researchers from discerning their basis and potentially overlooking crucial information. Researchers then gain little insight into the motivations driving respondent’s closed answer responses and are left to hypothesize as to the reasons on their own or include additional survey items and use more survey time. Yet, researchers who make and deploy their own survey on affective polarization have an opportunity to gain insight into respondent’s motivations directly from the respondents themselves. Accepting the expedient tradeoff is often prudent to conducing practical research and existing measures have many useful applications. However, an ideal measure is one that provides researchers with both the expediency of an easy-to-use scale and a method for extracting the motivations underlying respondents’ affect. This measure is our contribution: an easy-to-use survey instrument that captures respondents’ affective evaluations quantitatively and qualitatively, addressing problems that plague existing affective measures.

As opposed to closed-answer responses, which may be easier to analyze but can suffer from measurement error or internal validity issues [5, 20], we rely on open ended questions. By doing so, we allow respondents to explain their state of mind unrestricted by an artificially generated scale. To avoid the pitfalls of open-ended text data we rely on respondent self-coding [20]. While our approach is novel in measuring affective polarization, we build on previous scholars who use open-ended responses to generate quantitative data. For instance, Roberts et al. (2014) [21] use a structural topic model to classify the topics of open-ended survey questions using an out-of-the-box topic classifier. In addition, we draw on Glazier, Boydstun, and Feezell (2021) [20], who highlight the advantages of respondents coding their own open-ended responses. They demonstrate that these “self-coded evaluations” can mitigate concerns about intercoder reliability and reduce the bias that may occur when researchers code respondents’ open-ended answers. Our work also builds on Zollinger (2024) [22], who explores in-group and out-group identity formation based on voters’ qualitative responses. We ask respondents to provide us with one word to describe in and out partisans, while Zollinger (2024) [22] asks voters to describe a portrait of ingroup and out group partisans. While Zollinger (2024) [22] methodology is distinct from ours, we both aim to identify the cleavages that separate voters in the political parties we investigate through qualitative responses. Our work builds on research of scholars who devise creative methodologies for extracting quantitative information from qualitative survey response [23–25]; in addition to research that outlines the perils of researcher coded open-ended survey questions [26, 27].

We base our measure in the Receive-Accept-Sample (RAS) model of public opinion formation [6]. Specifically, Zaller’s theory of survey responses which posits that, rather than walking around with fixed opinions, respondents construct answers to survey questions when prompted. Survey responses are generated by sampling top-of-mind considerations. The RAS model’s Response axiom posits that, in answering survey questions, respondents average salient considerations to construct an answer. Furthermore, because the salience of considerations changes over time, each respondent’s answer represents a draw from a probability field of possible answers. The RAS model’s main contribution is in explaining response instability over time. However, we leverage the cognitive process proposed in *The Nature and Origins of Mass Opinion* [6] to measure a part of the opinion construction process.

**Fig 1:**
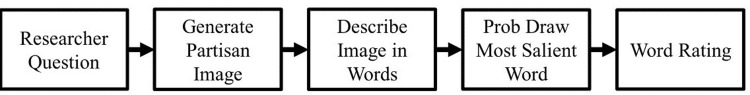
Model of response generation.

**Fig 2:**
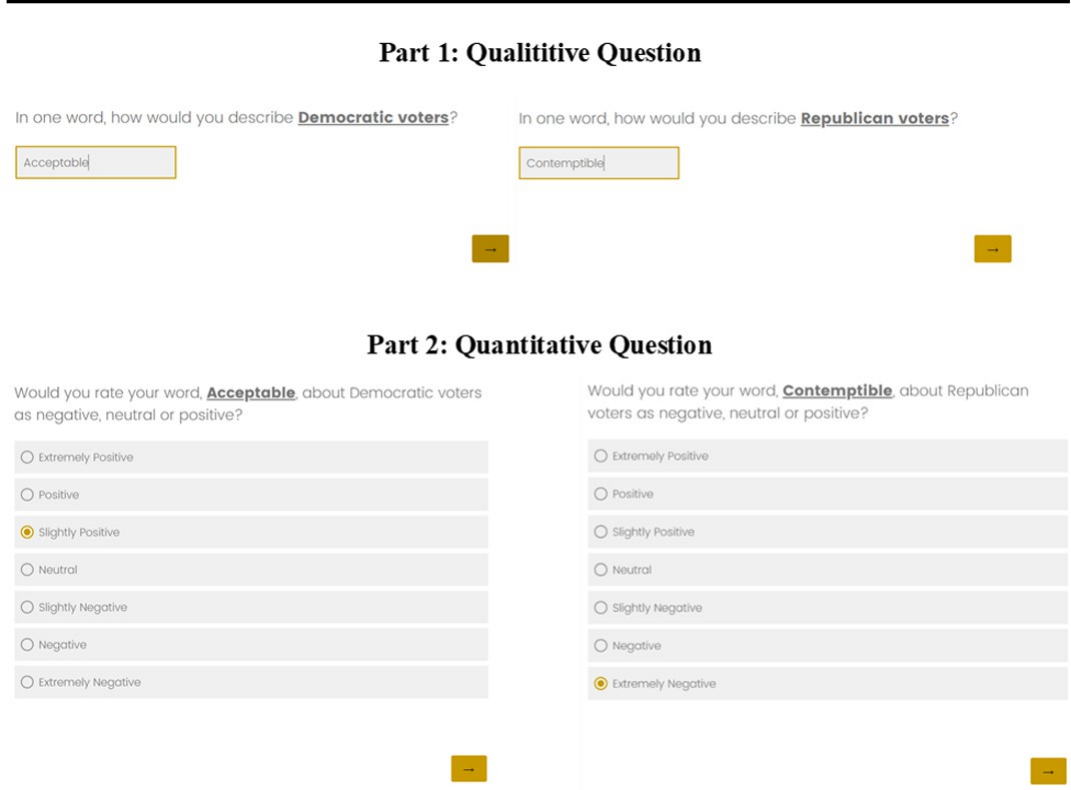
One word survey question example.

When prompted with any survey question asking respondents to evaluate out partisans, an answer will be constructed when the question is posed. Respondents will generate an image of a partisan, their characterization of the group, based in salient considerations at the time. Then respondents will necessarily have to describe this image and characterize it in words. Our measure prompts respondents to provide researchers with the most salient word in their mind used to describe the group being asked about. We then ask them to provide us a numerical rating. This process is shown in a flow chart in Fig 1 below while an image of our survey instrument is shown in the following section in Fig 2.

Note that, according to the RAS model, this process would unfold similarly for a thermometer score. Respondents would be prompted by the question to create an image of partisans based in salient considerations. They would then have to think about that image in terms of language. However, thermometer scores only have respondents provide a numerical rating for their feelings towards the summation of salient considerations regarding partisans. They provide no insight into the image or language a respondent uses to characterize the group in question. This does not mean thermometers are not perfectly valid measure. Indeed, they have widespread applications and uses as a solid workhorse measurement tool. However, if a researcher wants more detailed insights into respondents’ considerations, they are faced with the difficulty of working with unwieldy open-ended data. Should a researcher desire increased detail while avoiding the complexities and potential burdens associated with extensive open-ended responses, there is a need for a novel methodological approach that provides a balance between depth and practicality.

Building on current literature, our new measure of affective polarization has four major advantages over existing measures. First, it provides a more direct measurement of respondents’ affect by eliciting top of mind natural language considerations rather than constraining respondents to a scale. Second, by utilizing respondent self-scoring, we eliminate the difficulties associated with quantifying open ended responses [20]. Third, the new measure captures a distinct aspect of affect, as it produces similar predictive estimates to existing measures but is not perfectly correlated. Finally, one-word responses provide qualitative value that is useful for defining the motivations behind affective polarization that scholars propose in theory and that we show in this paper.

## Methodology

In the summer of 2021, we conducted a survey asking respondents for their one-word evaluations of in and out-partisans. Our survey yielded more than 1,300 high-quality and nationally representative respondents recruited using Lucid’s survey platform. To construct our new measure we first asked respondents to provide qualitative data by describing Democrats and Republicans using only one word. Then, we ask respondents to self-code the sentiment behind their word choice on a 7-point scale ranging from “extremely negative” (-3) to “extremely positive” (3), with “neutral” (0) as the midpoint. Fig 2 shows two examples of how a respondent might engage with our question.

We prompt the respondent to provide one word that describes Democratic and Republican voters. The first question represents the qualitative portion of our measure where we ask respondents to consider their feelings and express them through natural language. This engagement offers researchers important information about the reasons behind a respondent’s affect. The follow-up question allows the respondent to self-code the sentiment behind their word choice. This process takes the sentiment coding out of the researchers’ hands, empowering the respondent to provide the affect behind their word choice.

**Fig 3:**
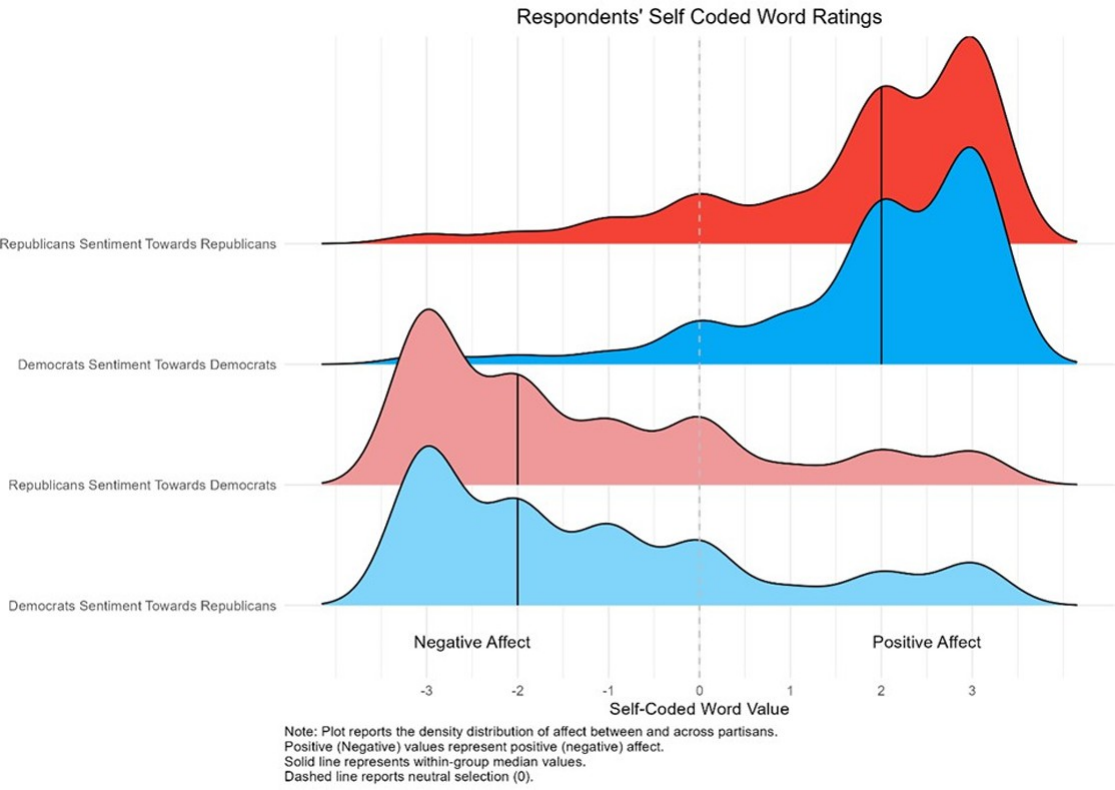
Distribution of respondents’ self-coded word on a seven-point scale.

Importantly, we do not ask respondents to code their affective feelings, but to code the word that comes to their mind when we prompt their affective feelings, making our measure of affect distinct from existing measures. Our goal is to quantify the sentiment behind their attitudes, not simply their attitudes themselves. While attitudes are more general evaluations, they are not necessarily rooted in emotional evaluations in the same way that sentiments are. An attitude may be a general belief, but a sentiment is a general reaction. Our two-part question prompts this reaction. If affect is rooted in emotional evaluations, then quantifying sentiment should better capture these evaluations than measuring beliefs. Fig 3 illustrates the distribution of respondents’ self-coded word on our seven-point scale. The figure reports partisans’ sentiment about both in and out-groups.

First, both Democrats and Republicans have positive affect toward individuals sharing their party identification. 85 percent of Democrats and 81 percent of Republicans chose words that they coded as positive. When describing their in-group, Democrats and Republicans have the same median sentiment score, 2, and similar in-group sentiment averages 1.9 and 1.8, respectively. Second, Democrats and Republicans have negative affect toward individuals with an opposing party identification: 68 percent of Democrats and 69 percent of Republicans coded their out-party word negatively. The median sentiment score was -2 and the average sentiment scores were -1.1 for Democrats and -1.2 for Republicans describing members of the out-party. *Prima facie*, our open ended survey responses follow a distribution that current literature might expect: strong emotional affect in favor of one’s own party and against their out-party [2, 16].

To validate our one-word measure, we focus on providing evidence that our one-word evaluations and their self-code have strong internal and external validity. Therefore, in the sections to follow we illustrate internal validity in two ways. First, we look at the words respondents report and the accompanying codes. Second, we compare the self-coded one-word responses to the most common measures of affect researchers use in the literature. Our goal is to show that our measure of affect has high face validity, high internal validity, and uncovers motivations underlying affective polarization that existing measures cannot. The following sections conducts two tests to evaluate the validity of our one-word affect measure. To start, we provide evidence showing self-coded one word evaluations measure respondents’ *affect* toward in-partisans and out-partisans. Then, we examine how well our one-word measure compares with existing affect measures. Finally, we provide evidence of the post collection value of text data produced by one-word measures by exploring the dimensions of affect and showing systematic differences in polarization between respondents who used policy vs valance words.

## Internal validity

We begin our internal validity exercise by looking directly at the open-ended words that individuals report about their feelings towards in and out partisans. These words validate our measure and capture the emotional responses driving respondents’ affective feelings towards each group. We find that individuals’ chosen words and codes display meaningful affect.

**Fig 4:**
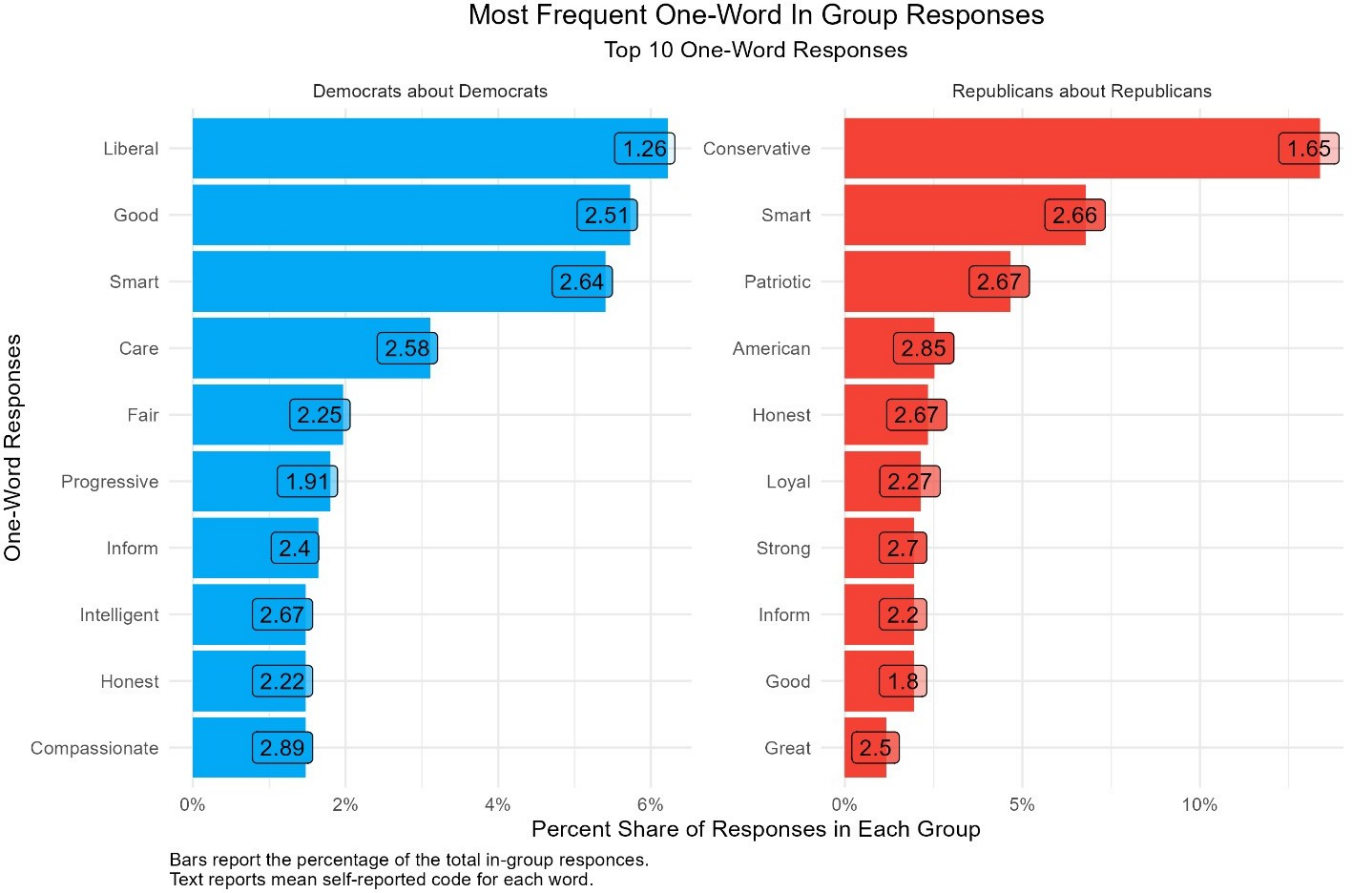
Distribution of the most common words about in-partisans.

**Fig 5:**
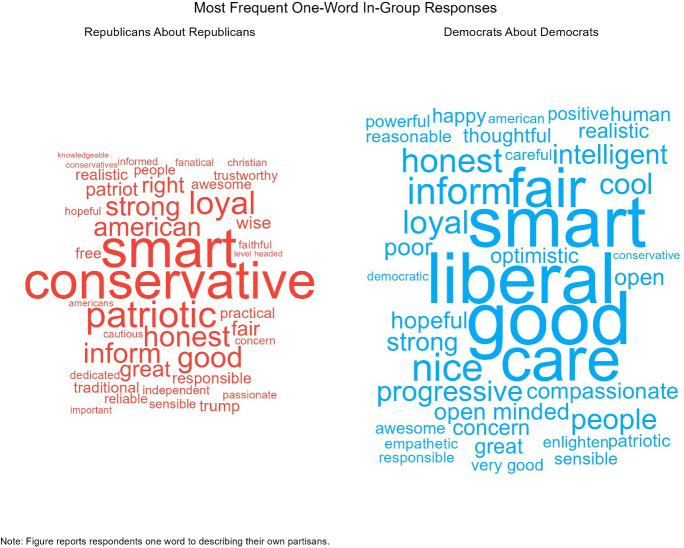
Word cloud of the most common words about in-partisans.

Figs 4 and 5 shows the distribution of the most popular words that Democrats and Republicans reported about their in-partisans and the average self-coded responses for each word. In each case, the plurality words partisans report about their own group are ideological in nature. Around 6 percent of Democrats report the word “liberal” and 15 percent of Republicans report the word “conservative.” Yet, Fig 4 reports more clearly that an overwhelming majority of words tend to be affective in nature: Democrats characterize themselves as “smart,” “good,” “caring,” and “compassionate;” while Republicans characterize themselves as “smart,” patriotic,” “American,” and “good.” Additionally, when we ask respondents to code these words, the responses we receive are congruent with our expectations about respondents’ feelings towards members of their own party. On average, respondents’ self-coded word reports a positive evaluation of in-partisans.

**Fig 6:**
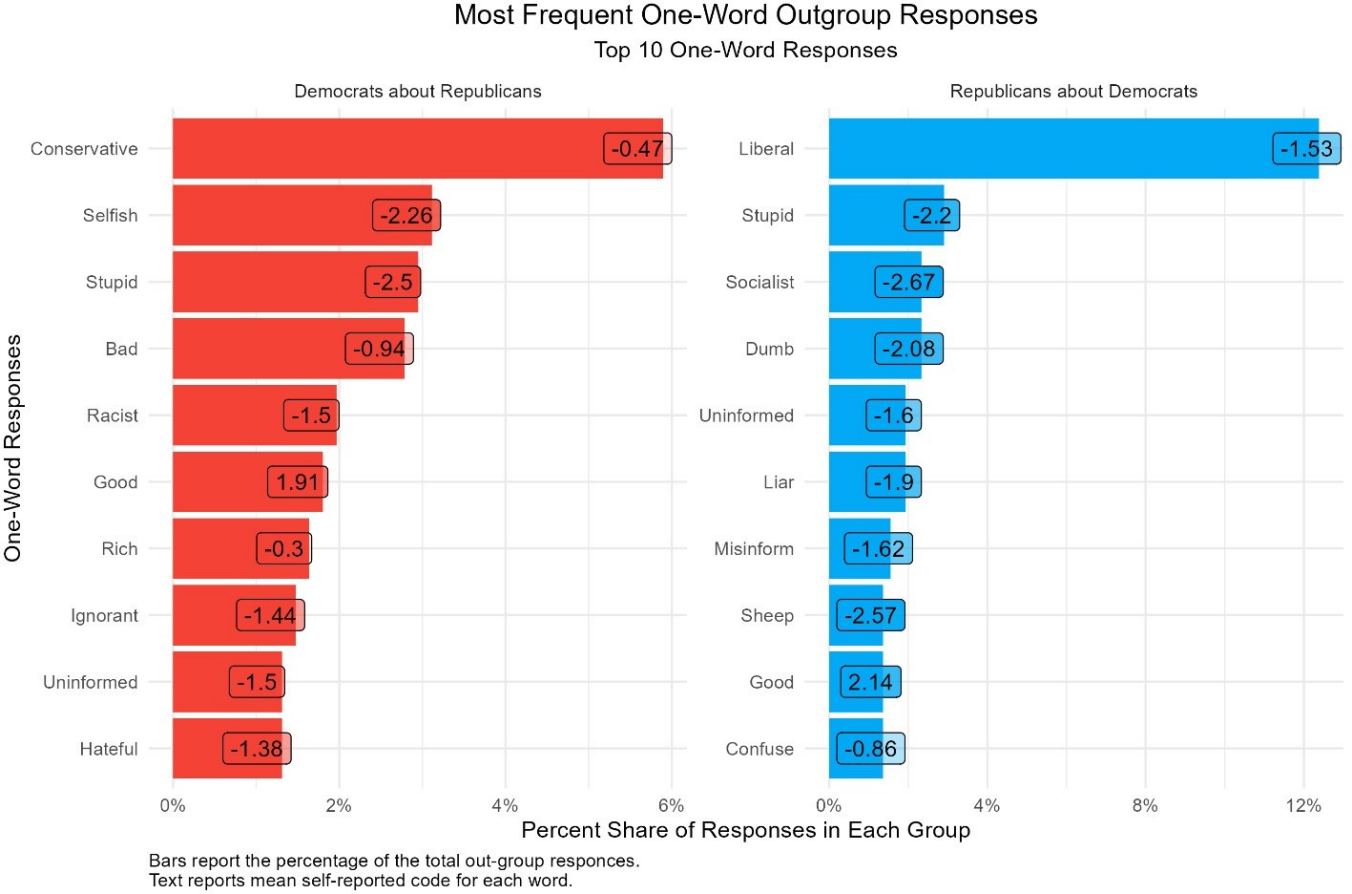
Distribution of the most common words about out-partisans.

Next, we find that out-group responses are even more affective. Figs 4 and 5 show the distribution of the most popular words that partisans report about out-partisans and the average self-coded response for each word. Like the previous results, the most common words are ideological in nature, “liberal” and “conservative.” However, Fig 6 shows that negative emotional evaluations of out-partisans account for a majority of the words. Democrats characterize Republicans as being “selfish,” “stupid,” “racist,” and “hateful;” while, Republican characterize Democrats as being “stupid,” “socialists,” “sheep,” and “confused”.

Importantly, when respondents code their word, the responses are congruent with our expectations about partisans’ feelings toward out-partisans. On average, Fig 6 reports that respondents select a word and code it as negative when asked to evaluate out-partisans.

**Fig 7:**
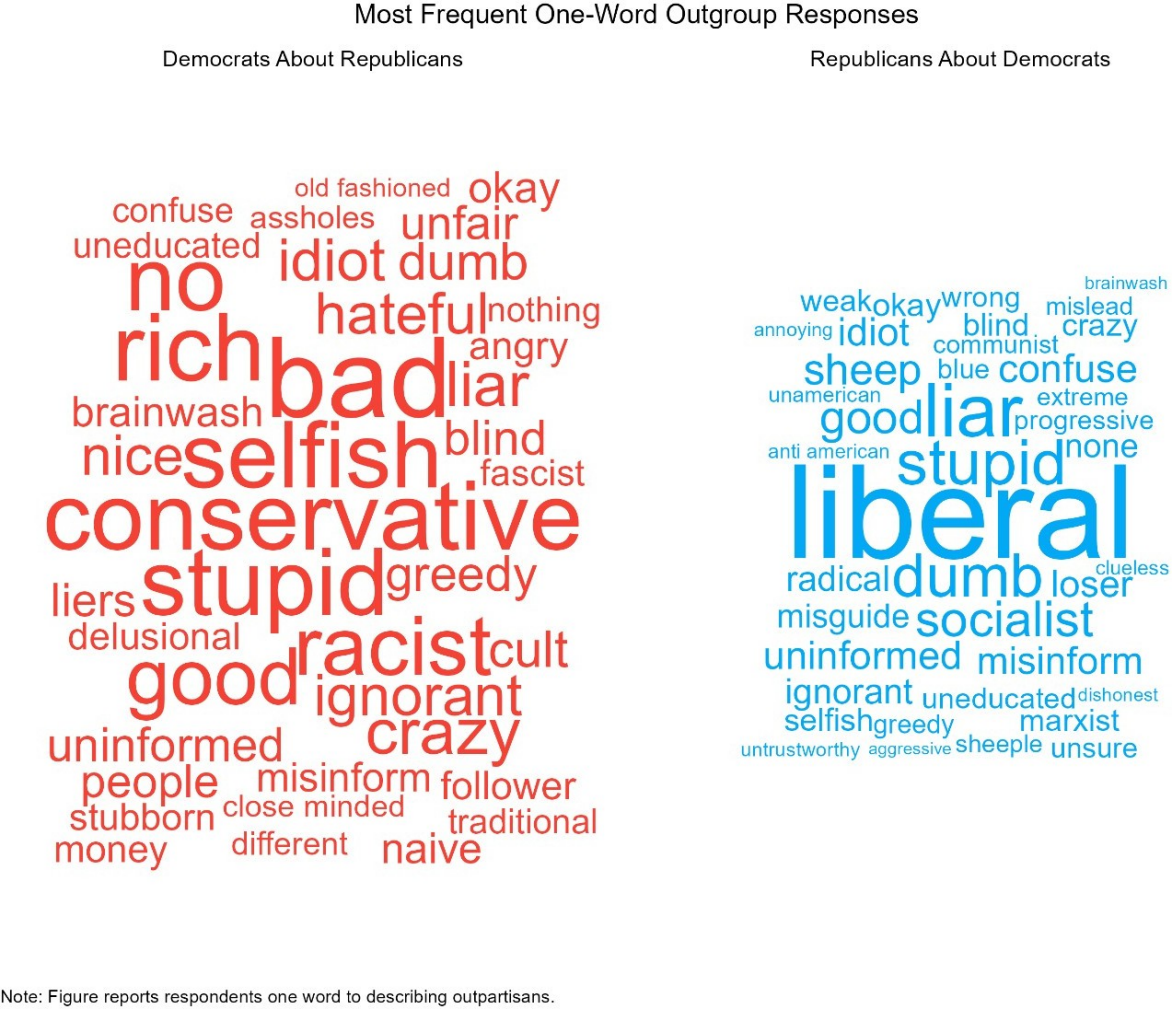
Word cloud of the most common words about out-partisans.

Together, these results suggest that the one-word evaluations and their subsequent codes have strong face validity. Respondents select words based on their emotional evaluation of in and out-partisans and the codes they assign are congruent with the literature’s expectations. Additionally, we can see in Fig 6 that our measure provides a novel insight into an asymmetry in levels of affective polarization. Among Democrats the main ideological word used to characterize the our party “Conservative”, has an average rating of -.47 suggesting a slightly negative evaluation. However, Republican respondents on average rate the main ideological word used to characterize the out party “Liberal” as -1.5 suggesting a highly negative evaluation. Our measure reveals that ratings of the parties’ main ideological words are asymmetrically polarized. Republicans view liberalism far more negatively than Democrats view conservatism. This distinction would be obscured with any other measure. For example, sentiment dictionaries applied to open ended answer would classify liberal and conservative as neutral, while closed ended answers would produce only the end point numerical values. Our measure shows that Republicans feel far more negatively towards the ideology of the Democratic party than Democrats do towards the ideology of the Republican party. See Fig 7 for a qualitative presentation of out-partisan sentiments.

### Comparison with existing measures

Next, we compare our one-word self-coded measure of affect with three well-established measures: thermometer scores, candidate evaluations, and a social-distance measure. We selected these measures as a benchmark because of their widespread use in the literature [2–4].

Researchers most frequently use thermometers to measure respondents’ affect [2, 3]. In our survey we asked respondents to rate their feelings about Democratic and Republican voters on a scale from 0 to 100. Then, to leverage trait ratings we use Lelkes, Sood, and Iyengar’s (2017) questions and average the scales together to create one measure for each party. Lastly, we create an affect measure based on Iyengar, Sood, and Lelkes’ (2012) [16] finding that respondents are unhappy seeing their son or daughter marry a member of the out-party. Building on this research, we included three questions in our survey asking how happy the respondent would be if their son or daughter married someone of the out-party, to live in a neighborhood composed of out partisans, and shop at a grocery store that contributed campaign contributions to out-partisan candidates [4, 28]. We average the scale together to create one measure for each party.

**Fig 8:**
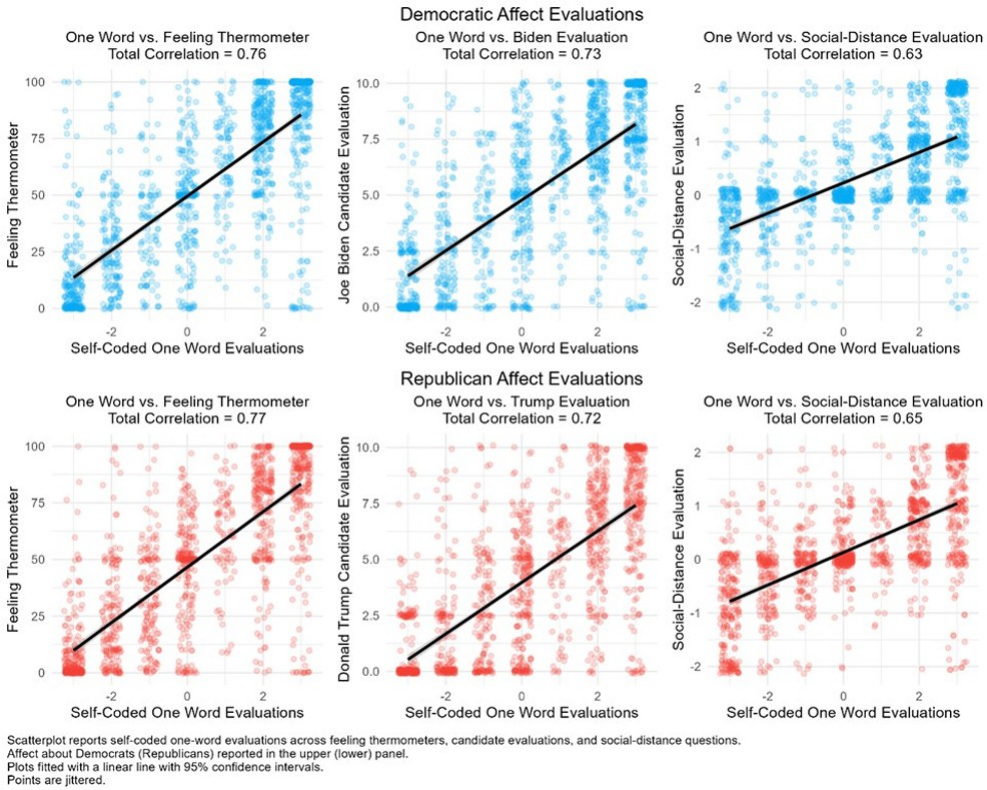
Scatterplot of self-coded one word evaluations vs existing measures.

When we compare respondents’ self-coded one-word evaluation of affect to the established measures, Fig 8 reports a remarkably strong relationship. First, when we compare one-word evaluations to Democratic and Republican feeling thermometers the correlations are 0.76 and 0.77, respectively. Second, the correlations between Democratic and Republican one-word evaluations and candidate evaluations of Joe Biden and Donald Trump are 0.73 and 0.73, respectively. Lastly, the correlations between Democratic and Republican one-word evaluations and lifestyle evaluations are 0.63 and 0.65, respectively. Together, the correlations report a strong relationship between one-word evaluation and established measures of affect.

While the correlations between the one-word evaluations and established measures are strongly related, they are not perfectly correlated; this is where their value lies. The residual correlation suggests our measure captures something unique about the latent dimension that current measures do not. In fact, we should be concerned if these measures were very strongly related because it would suggest that the one-word measure does not make a meaningful contribution.

To truly illustrate how our approach stands out from existing affect measures we need to leverage the words provided by respondents. Next, we examine the sematic undertones provided by responses and argue that our one-word measure captures distinctions between policy and valence as drivers of affective polarization. We explore these dimensions in the next section.

### Dimensions of affect

Current measures of affect, like thermometers, social-distance measures, and candidate evaluations [4], do an excellent job quantifying the divide between partisans. However, they fall short in their ability to qualitatively uncover heterogeneous motivations for respondents’ affective evaluations and dislike of their out-party.

Literature on emotional evaluations suggests that affect is based on subjective interpretations of the world [29]. These interpretations originate from a multidimensional structure [30], and inform how we evaluate the world around us [31]. Current measures of affective polarization reduce affective expressions to a single number and omit useful information about the motivations behind the respondent’s affective evaluations. This section leverages the content behind individuals’ one-word selection to define dimensions of respondents’ affect that we anticipate contribute to affective polarization in the political system and that have yet to be codified by previous literature. We contribute to the literature on affective polarization by defining and testing two dimensions that may underpin individuals’ affective evaluations of out-partisans. This novel property of our measure adds a different perspective to our understanding of affect and its role in American politics.

We hypothesize that two key motivations drive respondents’ affective evaluations of out-partisans and contribute to affective polarization in the United States: policy and valence. On the one hand, partisans’ animus between Democrats and Republicans may be the result of policy and ideological differences. Evidence suggests that the mass public is ideologically divided [32], that the public sees their world through a partisan lens and interpret the world, even basic facts, differently depending on their party identification [33, 34]; while policy divisions at the elite level trickle down to the voters who follow their lead [35].

On the other hand, partisans’ animus between Democrats and Republicans may be the result of negative character evaluations of the out-party [36, 37]. A respondent may contrast the policy divisions between themselves and an out-partisan and then ascribe a character (or valence) attribute onto an out-partisan because of their beliefs. For example, a Democrat might view a Republican’s anti-abortion stance as fundamentally opposed to their own belief in reproductive rights, leading them to attribute negative character traits, such as being oppressive or misogynistic, to the Republican. This theory is based in Tajfel and Turner (1979) [38] who define conditions that produce intergroup conflict based on an in-group ascribing negative character evaluations to an out-group they perceive as inferior.

To examine the policy and valence dimension of affective polarization, we hand-code code respondents’ one-word evaluations into three groups: neither policy nor valence, policy, or valence. The purpose of this exercise is to quantitatively assess the dimensions of a respondents’ affect towards in and out partisans. As our theory proposes, we anticipate that respondents’ affect is driven by either policy or valence. Therefore, we can better understand the respondents’ visceral reactions, gut response, and top-of-bucket state-of-mind affect by coding each one-word response.

We apply the following coding rules to each category of words. First, a policy word is any word that talks about policy or has an ideological direction to it. We code words like “liberal,” “conservative,” “socialist,” and “fascists” as policy. Second, a valence word is any word that talks about demeanor, behavior, or character [36, 37]. We code words like “stupid,” “uninformed,” “sheep,” “hateful” and vulgar characterizations as a valence evaluation. Additionally, we also code words ascribing positive evaluations like “smart,” “correct,” “intelligent” and “good” as valence as well. Lastly, we code words that neither directly describe character or have a policy or ideological angle to them as “neither policy nor valence.” These include words like “voter,” “money,” and “workers.” We represent the valence and policy dimension as two dummy variables that serve as our key independent variables, with words representing neither policy nor valence serving as the reference category. Together, our independent variable represents the two dimensions of affect, valence and policy, that respondents rely on to evaluate voters. In the model, we only use out-party evaluations to create these two dimensions. In the appendix, we report the full results of the models using in-party evaluations to create these two dimensions in Tables 1A-4A in S1 Appendix. We use these independent variables to predict the affective polarization scores using our respondent-coded one-word evaluation.

Affective polarization scores serve as our dependent variable. We create our measure of affective polarization by applying the same formula scholars use to create thermometers, candidate evaluation, and social-distance affective polarization to the respondent’s self-coding of their one-word evaluation:

AffectivePolarization=InPartyEvaluation−OutPartyEvaluation

The formula for affective polarization uses respondents’ self-coded word and takes their in-party affect evaluation and subtracts it from a respondents’ out-party affect evaluation. The formula generates a score ranging from -6 representing extreme in-party dislike to 6 representing extreme out-party dislike, with 0 indicating indifference between both parties. As a theoretical extension, we also use the policy and valence dimension to predict affective polarizations using thermometers, candidate evaluation, and social-distance measures.

We employ an ordinary least squares model that regresses affective polarization (using each measure of interest) onto our policy and valence dimension using the following formula:

$$\begin{array}{l} Affective\;Polarization\;\\ =\alpha +\beta_{1}\;(Policy\;Dimension)+\beta_{2}\;(Valence\;Dimension)+\beta_{i}\;\left(X_{i}\right)+\delta_{s}+\varepsilon \end{array}$$

*Affective Polarization* represents the values of affective polarization derived using each measure (self-coded words, thermometers, candidate evaluation, and social-distance). We standardize each measure of affective polarization so that mean is equal to 0 and the standard deviation is 1 to interpret the coefficients on the same scale. *Policy Dimension* represents words that have a policy or ideological meaning; while the *Valence Dimension* covers any words that have a positive or negative character evaluation. Together, these variables represent our key independent variables, and we compare their coefficient to the base term: non-policy or valence words.

*X*_*i*_ represents our control variables. Our controls include the respondents *self-coded one-word evaluation* of out-party voters. This control is the most important because it tests whether the valence or policy dimensions contributes predictive power to affective polarization, beyond merely the positive or negative evaluation of the word. Our model also controls include demographic characteristics such as the respondents’ *age*, *income*, *gender*, *education* and *ethnicity*. We also include terms measuring extremism in respondents *party identification* and *ideology*. Finally, we include political engagement measures: whether the respondent *donated* to a political candidate and whether they *voted* in the 2020 election.

We report the results of our key independent variable across three model specifications: base model (includes only our dependent and independent variable), control model (includes our controls along with our independent variable) and a state fixed effects model (*δ*_*s*_). We report the tables for each full specification across each affective polarization measure in Tables 1A-4A in S1 Appendix. We also report alternative model specifications in Fig 3A in S1 Appendix.

**Fig 9:**
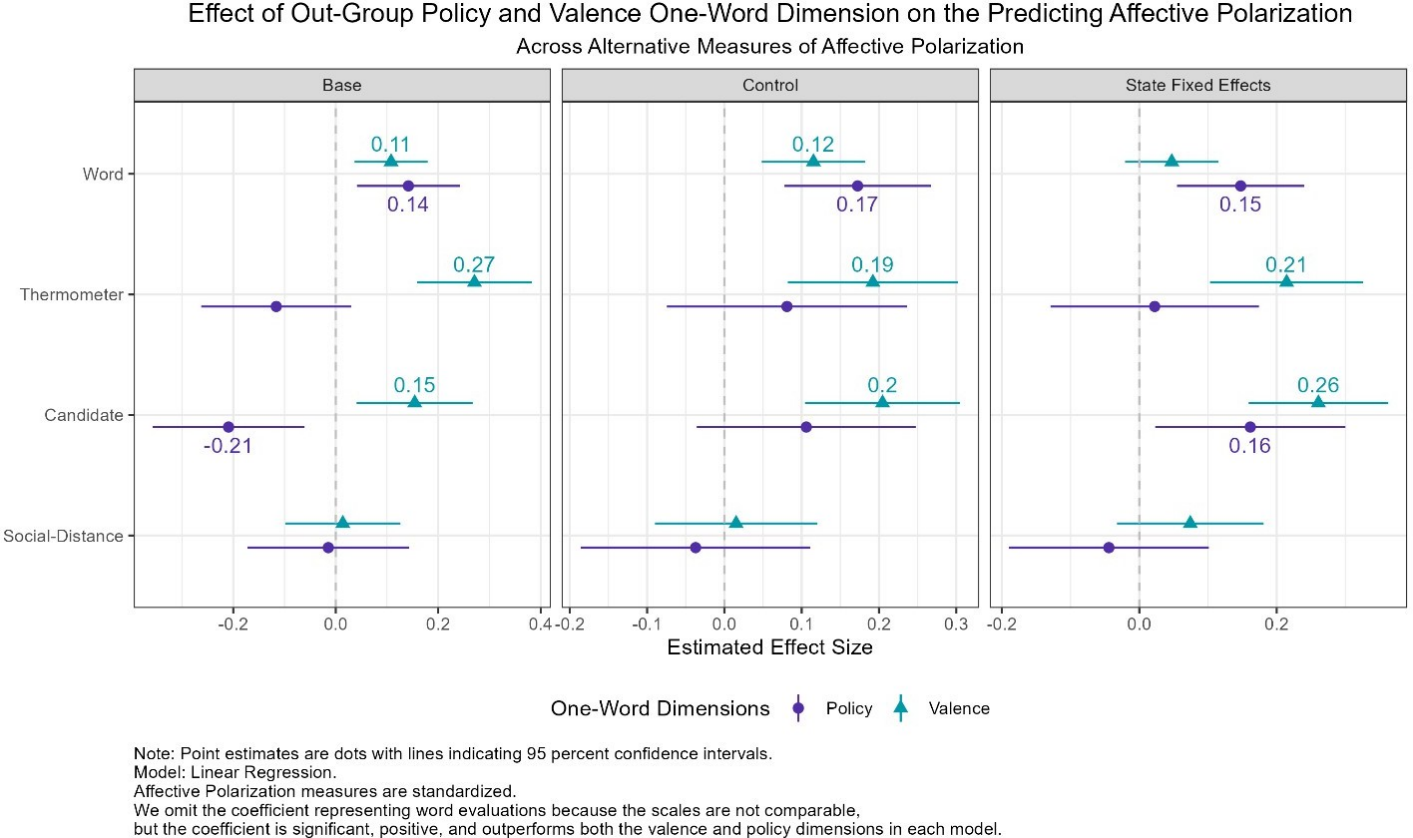
Estimates for the effect of out-group policy and valence dimension on affective polarization.

We find that the valence dimension contributes to predicting affective polarization. Fig 9 reports the results of the linear model regressing the affective measures onto the policy and valence dimensions. When predicting affective polarization using the self-coded one-word evaluations, the valence and policy dimension preform equally as well (positive and statistically significant), with the policy dimension outperforming in the state fixed effects model. When predicting affective polarization using thermometer scores or candidate evaluations, the valence dimension outperforms the policy dimension. Finally, nether the policy nor valence dimensions are useful beyond the positive or negative one-word evaluations in predicting affective polarization using social distance measures. In each model, the coefficient representing respondents’ one-word evaluations of out-partisan voters is significant, positive, and outperforms both the valence and policy dimensions in each model. Yet, across key indicators of affective polarization, both the valence and policy dimensions contribute additional predictive power that suggests that valence and policy evaluations of out-partisans may motivate affective polarization.

Taking the control model, we find that when compared to non-policy or valence words, valence words increase affective polarization as measured using words, thermometers, and candidate evaluations measures by 0.12, 0.19, and 0.2 units, respectively. Meaning that valence evaluations of the out-party increase affective polarization by a fifth to at least a tenth of a standard deviation.

Consequently, even when we include respondents’ one-word evaluations of out-partisans into the model, the valence dimension is a significant predictor of affective polarization. This effect is meaningful because it shows that the valence dimension exerts an effect that is *independent* from the positive/negative sentiment of the word alone. Therefore, the *content* of respondents’ affect (i.e., character evaluation) is meaningful in understanding affective polarization. This additional content is a key contribution of our one word measures.

These results report that character evaluations of out-partisans are associated with greater affective polarization than policy or ideological assessment of the out-party. The results imply that the partisan animus behind affective polarization is not solely rooted in policy but also in the character evaluations that partisan project onto their counterpart. While not unique in this regard, these finding do further our understanding of how negative character evaluations drive the distrust and dislike that partisans harbor for one another. Indeed, our findings fit with and connect to a broader literature on discursive polarization in communication between partisans [39]. Other scholars, such as Bruggemann and Meyer (2023) [39], have found that out group communication is characterized by hostile and dismissive interactions at least partially driven by negative constructions of the outgroup [39]. These findings should raise the alarm that policy gridlock, media characterizations, and firebrand rhetoric fundamentally reveal divisions among partisans that go well beyond rational policy dimensions but embed themselves in the emotional consciousness of partisans.

These results are striking because they reveal that established quantitative measures of affect contain key information about the nature of respondents’ affect, which we show that one-word evaluations help to clarify. Without the qualitative assessment that one-word answers offer, scholars may be missing key variation in respondents’ affective attitude about out-partisans obscured by affective polarization scores. Here our qualitative exercise highlights the character dimensions that comprise the emotional affective dimensions that explains affective polarization. Further, these results lend support to Tajfel and Turner’s (1979) [38] social identity theory that the literature has taken as true and we formalize in our analysis.

In sum, these results show that dimensions of respondent’s affect towards out-partisans contribute to our understanding of affective polarization. Particularly, respondents harboring negative character evaluations tend to have higher levels affective polarization, as measured by words, thermometers, and candidate evaluations. These dimensions contribute *independently* to respondent’s one-word evaluations and makes them unique in explaining affective polarization.

## Conclusion

As political divisions expand, our need to measure affect and polarization in the public becomes even more valuable. However, while extremely useful, current measures of affect, such as thermometers scores, candidate evaluations, and social-distance measures are closed-ended in their nature. To better measure these divisions, we introduce a unique measure of affect that draws on innovations in open-ended responses. Our open-ended approach measures partisan affect by allowing respondents to provide their top-of-mind evaluation, without the restrictions of a bounded scale. Then, having respondents self-code their answer provides researchers with an easy-to-use scale the retains the rich information open ended responses provide.

If scholars aim to capture *affective* evaluations, then we argue that the measures we introduce captures respondents’ *emotional* evaluations about partisans; thereby enhancing the *validity* of affective analyses. We support our argument through a battery of internal and external validity checks. First, we report the raw words that respondents selected and show face validity in their emotional evaluations of in and out-partisans ("F*cktards” and “Poopybutts”). Second, we show that our new measures are highly, but not perfectly correlated, to established measures of affect [4, 16]. These results imply that our measures capture a unique feature of affect that existing measures may not. This unique feature better captures the *sentiment* behind their *emotional* evaluations.

Together, our analysis suggests one-word evaluations contribute to our understanding of partisan affect and affective polarization by revealing the motivations behind respondents’ evaluations. The type of analysis presented here can only be done using self-coded one-word responses since traditional measures reduce emotional responses onto a bounded scale and fully open-ended responses are too unwieldy for quantitative analysis.

Despite our efforts, our protocol it not without limitations. First, unless self-coding open-ended responses are widely implemented in major national surveys (CCES/ANES), only researchers who have control over the questions in their own survey can implement self-coding. This is a solvable problem. If CCES and ANES, introduce self-coded one-word responses, then scholars can use open-ended responses or validate existing affect measures. Second, our survey item involves respondents typing a response and then coding it. This two-step process takes additional time compared to traditional scales. This cost is unavoidable. Yet, we believe the benefits outlined above are well worth the survey time. Third, this paper uses one word and one self-code of that word to measure affect. In effect, this approach asks respondents to take complex human emotions and motivations and summarize them into a single word, which may be seen as being reductionist. Methodologically, it may miss out on the opportunity to obtain deeper insights into the respondents’ motivations by inviting them to share more detailed qualitative insights. Longer open-ended text fields, qualitative interviews, and focus groups may serve as useful alternatives to a single word and result in collecting more comprehensive qualitative data. While our method introduces a new survey protocol to affective polarization research, we recognize that it might not provide the depth of insight achievable through more expansive qualitative techniques. Therefore, leveraging a self-coding protocol alongside deeper quantitative analysis could powerfully enable researchers to construct a qualitative dataset, converting them into quantitative insights, and test hypotheses about respondents’ emotions, attitudes, or motivations. Irrespective of the quantitative approach, allowing respondents to self-code their qualitative responses enrich the dataset with nuanced insights directly from the source, enhances the accuracy, usefulness and relevance of the data collected. We encourage future researchers to pair our self-coding procedure with a longer qualitative response from participants to gain the benefits of self-coding along with richer qualitative insights.

While our measure relies on a self-coded evaluation, alternative methods to quantify open-ended responses exist. For example, large language models and natural language processing are growing in popularity due to their ability to process large amounts of text and evaluate it across various dimensions [23, 24, 40]. These models can classify underlying sentiment (positive, neutral, or negative) [41] and emotion (anger, sadness, joy, etc.) [42], identify people, places, or things, and group common themes together across responses [43]. These processing tools are powerful, particularly for text that have already been collected by researchers, text that the author cannot evaluation themselves, or long-form open-ended survey responses that researchers want to quantify. Text processing tools with these capabilities are becoming increasingly democratized. Free and open-source text processing tools are available on sites like GitHub and Hugging Face. While proprietary LLM’s like OpenAI’s ChatGPT, Google’s Gemini, Anthropic’ Claude 3 and more are becoming cheaper, more affordable, and more accessible every year. For researcher who are interested in getting started immediately, we believe that they should leverage ChatGPT as a revolutionary text classification tool [44–46]. Through prompt engineering, researchers can use ChatGPT as a research assistant to analyze survey responses, classify respondents’ emotions and sentiments, and extract common themes within a text corpus. Leveraging these advancements in natural language processing, particularly ChatGPT, researcher can take in long-form qualitative data and derive quantitative measures efficiently and accurately.

Despite limitations and alternative classification methods, for scholars looking to move forward with our survey protocol, we have several practical avenues for future research. First, scholars can apply our protocol comparatively to study affective polarization partisans in other counties. Whether there are cross-national differences in the dimensions of partisan affect is a question our protocol is well suited to answer. Additionally, differences in how partisans characterize each other across parties in multi-party democracies is likely a promising area of research given existing work on the topic [11]. Second, scholars can use our protocol to measure divisions between any group of individuals. For example, Amlani and Kiesel (2022) [47] examine one word evaluations of vaccinated or unvaccinated Americans. Third, we asked respondents to code the sentiment of their word; however, scholars can also ask respondents to choose from a preexisting list of emotions (i.e., angry, frustration, sad, or happy). This additional step would provide scholars with a quantitative understanding of the emotions that underlie respondents’ choice of words.

## Supporting information

S1 Appendix (DOCX)
