# Supplementary material for: Affective polarization in a word: Open-ended and self-coded evaluations of partisan affect
Source: PLoS One. 2025 Jan 16;20(1):e0310772. doi: 10.1371/journal.pone.0310772 (PMC11737763; doi:10.1371/journal.pone.0310772)
Supplement: S1 Appendix — (DOCX) [file pone.0310772.s001.docx]

**Supplement Material for**

“Affective Polarization in a Word:

Open-Ended and Self-Coded Evaluations of Partisan Affect”

January 12, 2024

Table of Contents

[Survey Questions 2](#_Toc130197962)

[Survey Quality 3](#_Toc130197963)

[Full Tables from Affect Dimensions Analysis 5](#_Toc130197964)

[Liberal and Conservative as Words Evoke Affective Responses 9](#_Toc130197965)

[Figure 1A: Distribution of Self-Coded “Liberal” and “Conservative” One-Word Evaluations 9](#_Toc130197966)

[Measures of Affective Polarization are Highly Correlated 10](#_Toc130197967)

[Figure 2A: Scatterplot of Self-Coded One Word Evaluations Against Existing Affective Polarization Measures 11](#_Toc130197968)

[Affective Word Dimensions Using In-Party Specification 11](#_Toc130197969)

[Figure 3A: Estimates for the Effect of In-Group Policy and Valence Dimension on Affective Polarization 12](#_Toc130197970)

[The Benefits of Open-Ended Responses With Self-Coded Words 12](#_Toc130197971)

[Work Cited 14](#_Toc130197972)

# Survey Questions

In one word, how would you describe Republican voters?

[Open-Ended Response 1]

Would you rate your word, [Open-Ended Response 1], about Republican voters as negative, neutral or positive?

- Extremely Positive
- Positive
- Slightly Positive
- Neutral
- Slightly Negative
- Negative
- Extremely Negative

In one word, how would you describe Democratic voters?

[Open-Ended Response 2]

Would you rate your word, [Open-Ended Response 2], about Democratic voters as negative, neutral or positive?

- Extremely Positive
- Positive
- Slightly Positive
- Neutral
- Slightly Negative
- Negative
- Extremely Negative

# Survey Quality

When fielding our survey on Lucid, we knew of the potential threat of receiving a low-quality sample. To ensure that our sample was high quality, we implemented checks into the fielding process. These checks include three attention check questions (one of which dismissed respondents who failed), timing questions that dropped individuals who took too long or sped through the survey too quickly, and missing questions check that dropped respondents who left too many missing questions.

Our survey includes three attention check questions. The first attention check question is as follows:

*For our research, careful attention to survey questions is critical! We thank you for your care.*

*I understand*

*I do not understand*

We placed this question at the beginning of the survey. Respondents who selected *I do not understand* were dropped in the sample.

The second attention check question is as follows:

*For our research, careful attention to survey questions is critical! To show that you are paying attention, please select “I have a question” and "I understand" below.*

*I understand*

*I do not understand*

*I have a question*

*I do not have a question*

We dropped respondents who did not check both “I have a question” and "I understand" from the sample and replaced by lucid without charge.

The final attention check question was placed at the end of the survey and is as follows:

*People are very busy these days and many do not have time to follow what goes on in the government. We are testing whether people read questions. To show that you've read this much, answer both "extremely interested" and "very interested."*

*Extremely interested*

*Very interested*

*Moderately interested*

*Slightly interested*

We use this questions from (1) paper on crafting attention check questions for Lucid. We dropped respondents who did not select both *extremely interested* and *very interested* in the analysis.

Next, we dropped respondents who took less than 6 minutes and more than 40 minutes to complete the survey from the analysis.

Then, we dropped respondents who did not answer at least 50 questions (or at least 60 percent of the maximum number of questions) from the analysis.

Finally, in the regression analysis exploring the dimensions of affect we drop words that do not make sense.

# Full Tables from Affect Dimensions Analysis

Tables 1A to 4A report the full table results we represent using Figure 7 in the dimensions of affect section.

| **Table 1A: Regression Results for Affect Dimension on Affective Polarization**  **Word Measure** | | | |
| --- | --- | --- | --- |
|  | | | |
|  | Dependent Variable: | | |
|  |  | | |
|  | *Word Measure* | | |
|  | Base | Control | State Fixed Effects |
|  | (1) | (2) | (3) |
|  | | | |
| Affect Dimension: Valence | 0.108^***^ (0.036) | 0.115^***^ (0.034) | 0.047 (0.035) |
| Affect Dimension: Policy | 0.142^***^ (0.051) | 0.172^***^ (0.048) | 0.148^***^ (0.047) |
| Word Evaluation | 0.365^***^ (0.008) | 0.375^***^ (0.009) | 0.379^***^ (0.009) |
| Age |  | -0.004^***^ (0.001) | -0.005^***^ (0.001) |
| Income |  | 0.004 (0.003) | 0.003 (0.003) |
| Gender: Male |  | 0.036 (0.034) | 0.071^**^ (0.035) |
| Education |  | -0.024^**^ (0.010) | -0.017^*^ (0.010) |
| Ethnicity: Black |  | -0.041 (0.047) | -0.007 (0.051) |
| Ethnicity: Asian |  | -0.324^***^ (0.069) | -0.350^***^ (0.070) |
| Ethnicity: Native American |  | 0.033 (0.162) | -0.026 (0.172) |
| Ethnicity: Pacific Islander |  | 0.193 (0.473) | 0.171 (0.446) |
| Ethnicity: Other |  | 0.263^**^ (0.103) | 0.298^***^ (0.103) |
| Ideological Extremity |  | 0.080^***^ (0.015) | 0.070^***^ (0.016) |
| Partisan Extremity |  | 0.157^***^ (0.024) | 0.182^***^ (0.024) |
| Donor: Yes |  | 0.088^**^ (0.041) | 0.084^**^ (0.041) |
| Voter: Yes |  | 0.081^*^ (0.042) | 0.149^***^ (0.043) |
| Constant | -0.518^***^ (0.025) | -0.834^***^ (0.087) | -0.908^***^ (0.181) |
|  | | | |
| Observations | 1,132 | 1,124 | 1,119 |
| Adjusted R2 | 0.638 | 0.698 | 0.736 |
| Residual Std. Error | 0.576 | 0.544 | 0.556 |
|  | | | |
| *Note:*  ^*^p<0.1; ^**^p<0.05; ^***^p<0.01  Affect dimension and word evaluation use words on out-partisans.  We word reverse-code word evaluation, so positive values indicate negative evaluations. | | | |

| **Table 2A: Regression Results for Affect Dimension on Affective Polarization**  **Thermometer Measure** | | | |
| --- | --- | --- | --- |
|  | | | |
|  | Dependent Variable: | | |
|  |  | | |
|  | *Thermometer Measure* | | |
|  | Base | Control | State Fixed Effects |
|  | (1) | (2) | (3) |
|  | | | |
| Affect Dimension: Valence | 0.271^***^ (0.057) | 0.192^***^ (0.056) | 0.215^***^ (0.057) |
| Affect Dimension: Policy | -0.116 (0.075) | 0.081 (0.079) | 0.022 (0.077) |
| Word Evaluation | 0.247^***^ (0.014) | 0.233^***^ (0.014) | 0.236^***^ (0.015) |
| Age |  | -0.002 (0.002) | -0.002 (0.002) |
| Income |  | 0.006 (0.004) | 0.013^***^ (0.004) |
| Gender: Male |  | 0.144^**^ (0.056) | 0.202^***^ (0.057) |
| Education |  | -0.079^***^ (0.017) | -0.066^***^ (0.017) |
| Ethnicity: Black |  | -0.079 (0.078) | -0.059 (0.084) |
| Ethnicity: Asian |  | 0.452^***^ (0.114) | 0.509^***^ (0.114) |
| Ethnicity: Native American |  | -0.017 (0.266) | -0.294 (0.281) |
| Ethnicity: Pacific Islander |  | -0.365 (0.775) | -0.460 (0.728) |
| Ethnicity: Other |  | -0.324^*^ (0.169) | -0.378^**^ (0.168) |
| Ideological Extremity |  | 0.012 (0.025) | -0.052^**^ (0.026) |
| Partisan Extremity |  | 0.340^***^ (0.039) | 0.400^***^ (0.039) |
| Donor: Yes |  | 0.222^***^ (0.068) | 0.151^**^ (0.067) |
| Voter: Yes |  | 0.012 (0.069) | 0.111 (0.071) |
| Constant | -0.369^***^ (0.039) | -0.935^***^ (0.142) | -0.719^**^ (0.296) |
|  | | | |
| Observations | 1,123 | 1,115 | 1,110 |
| Adjusted R2 | 0.259 | 0.306 | 0.402 |
| Residual Std. Error | 0.859 | 0.833 | 0.865 |
|  | | | |
| *Note:*  ^*^p<0.1; ^**^p<0.05; ^***^p<0.01  Affect dimension and word evaluation use words on out-partisans.  We word reverse-code word evaluation, so positive values indicate negative evaluations. | | | |

| **Table 3A: Regression Results for Affect Dimension on Affective Polarization**  **Candidate Measure** | | | |
| --- | --- | --- | --- |
|  | | | |
|  | Dependent Variable: | | |
|  |  | | |
|  | Candidate Measure | | |
|  | Base | Control | State Fixed Effects |
|  | (1) | (2) | (3) |
|  | | | |
| Affect Dimension: Valence | 0.154^***^ (0.058) | 0.205^***^ (0.051) | 0.261^***^ (0.052) |
| Affect Dimension: Policy | -0.209^***^ (0.076) | 0.106 (0.072) | 0.162^**^ (0.071) |
| Word Evaluation | 0.244^***^ (0.014) | 0.196^***^ (0.013) | 0.207^***^ (0.013) |
| Age |  | 0.005^***^ (0.001) | 0.005^***^ (0.001) |
| Income |  | -0.012^***^ (0.004) | -0.012^***^ (0.004) |
| Gender: Male |  | 0.021 (0.051) | 0.057 (0.052) |
| Education |  | -0.059^***^ (0.015) | -0.069^***^ (0.016) |
| Ethnicity: Black |  | -0.140^**^ (0.071) | -0.252^***^ (0.076) |
| Ethnicity: Asian |  | 0.329^***^ (0.104) | 0.293^***^ (0.104) |
| Ethnicity: Native American |  | 0.688^***^ (0.243) | 0.724^***^ (0.256) |
| Ethnicity: Pacific Islander |  | 0.271 (0.708) | 0.423 (0.665) |
| Ethnicity: Other |  | -0.303^*^ (0.155) | -0.202 (0.153) |
| Ideological Extremity |  | 0.002 (0.023) | -0.025 (0.024) |
| Partisan Extremity |  | 0.187^***^ (0.036) | 0.214^***^ (0.036) |
| Donor: Yes |  | 0.158^**^ (0.062) | 0.147^**^ (0.061) |
| Voter: Yes |  | 0.163^***^ (0.063) | 0.229^***^ (0.065) |
| Constant | -0.310^***^ (0.039) | -0.680^***^ (0.130) | -0.335 (0.270) |
|  | | | |
| Observations | 1,132 | 1,124 | 1,119 |
| Adjusted R2 | 0.237 | 0.305 | 0.4 |
| Residual Std. Error | 0.872 | 0.876 | 0.893 |
|  | | | |
| *Note:*  ^*^p<0.1; ^**^p<0.05; ^***^p<0.01  Affect dimension and word evaluation use words on out-partisans.  We word reverse-code word evaluation, so positive values indicate negative evaluations. | | | |

| **Table 4A: Regression Results for Affect Dimension on Affective Polarization**  **Social-Distance Measure** | | | |
| --- | --- | --- | --- |
|  | | | |
|  | Dependent Variable: | | |
|  |  | | |
|  | *Social-Distance Measure* | | |
|  | Base | Control | State Fixed Effects |
|  | (1) | (2) | (3) |
|  | | | |
| Affect Dimension: Valence | 0.014 (0.057) | 0.015 (0.054) | 0.074 (0.054) |
| Affect Dimension: Policy | -0.015 (0.081) | -0.037 (0.076) | -0.044 (0.074) |
| Word Evaluation | 0.177^***^ (0.013) | 0.186^***^ (0.014) | 0.193^***^ (0.014) |
| Age |  | 0.002 (0.001) | 0.001 (0.002) |
| Income |  | 0.002 (0.004) | 0.005 (0.004) |
| Gender: Male |  | 0.059 (0.054) | 0.069 (0.055) |
| Education |  | -0.038^**^ (0.016) | -0.031^*^ (0.016) |
| Ethnicity: Black |  | 0.008 (0.074) | -0.003 (0.080) |
| Ethnicity: Asian |  | 0.147 (0.109) | 0.184^*^ (0.109) |
| Ethnicity: Native American |  | -0.182 (0.254) | -0.352 (0.270) |
| Ethnicity: Pacific Islander |  | -0.074 (0.742) | -0.051 (0.700) |
| Ethnicity: Other |  | -0.047 (0.162) | -0.105 (0.161) |
| Ideological Extremity |  | 0.171^***^ (0.024) | 0.128^***^ (0.025) |
| Partisan Extremity |  | 0.295^***^ (0.038) | 0.324^***^ (0.038) |
| Donor: Yes |  | 0.175^***^ (0.065) | 0.149^**^ (0.064) |
| Voter: Yes |  | -0.115^*^ (0.066) | -0.003 (0.068) |
| Constant | -0.189^***^ (0.039) | -1.041^***^ (0.136) | -0.477^*^ (0.284) |
|  | | | |
| Observations | 1,132 | 1,124 | 1,119 |
| Adjusted R2 | 0.138 | 0.281 | 0.377 |
| Residual Std. Error | 0.923 | 0.872 | 0.902 |
|  | | | |
| *Note:*  ^*^p<0.1; ^**^p<0.05; ^***^p<0.01  Affect dimension and word evaluation use words on out-partisans.  We word reverse-code word evaluation, so positive values indicate negative evaluations. | | | |

# Liberal and Conservative as Words Evoke Affective Responses

In the main text of the paper, we note that the words “liberal” and “conservative” are ideological but evoke affect among respondents. Figure 1A report a boxplot of Democrats and Republicans self-coding of the words “liberal” and “conservative” as it relates to their own party and the out-party. The figures illustrate that ideological words evoke affect among partisans.

When Democrats report “liberal” and Republicans report “conservative” to describe in-partisans, 72 percent of Democrats and 82 percent of Republican code their ideological word as having positive affect. On average, Democrats and Republicans report a self-coded evaluation equal to 1.19 and 1.63 (median equal to 1 and 2) respectively.

Contrastingly, when Democrats report “conservative” and Republicans report “liberal” to describe out-partisans, 82 percent of Democrats and 54 percent of Republican code their ideological word as having negative affect. On average, Democrats and Republicans report a self-coded evaluation equal to -0.45 and -1.52 (median equal to -1 and -2) respectively.

## Figure 1A: Distribution of Self-Coded “Liberal” and “Conservative” One-Word Evaluations


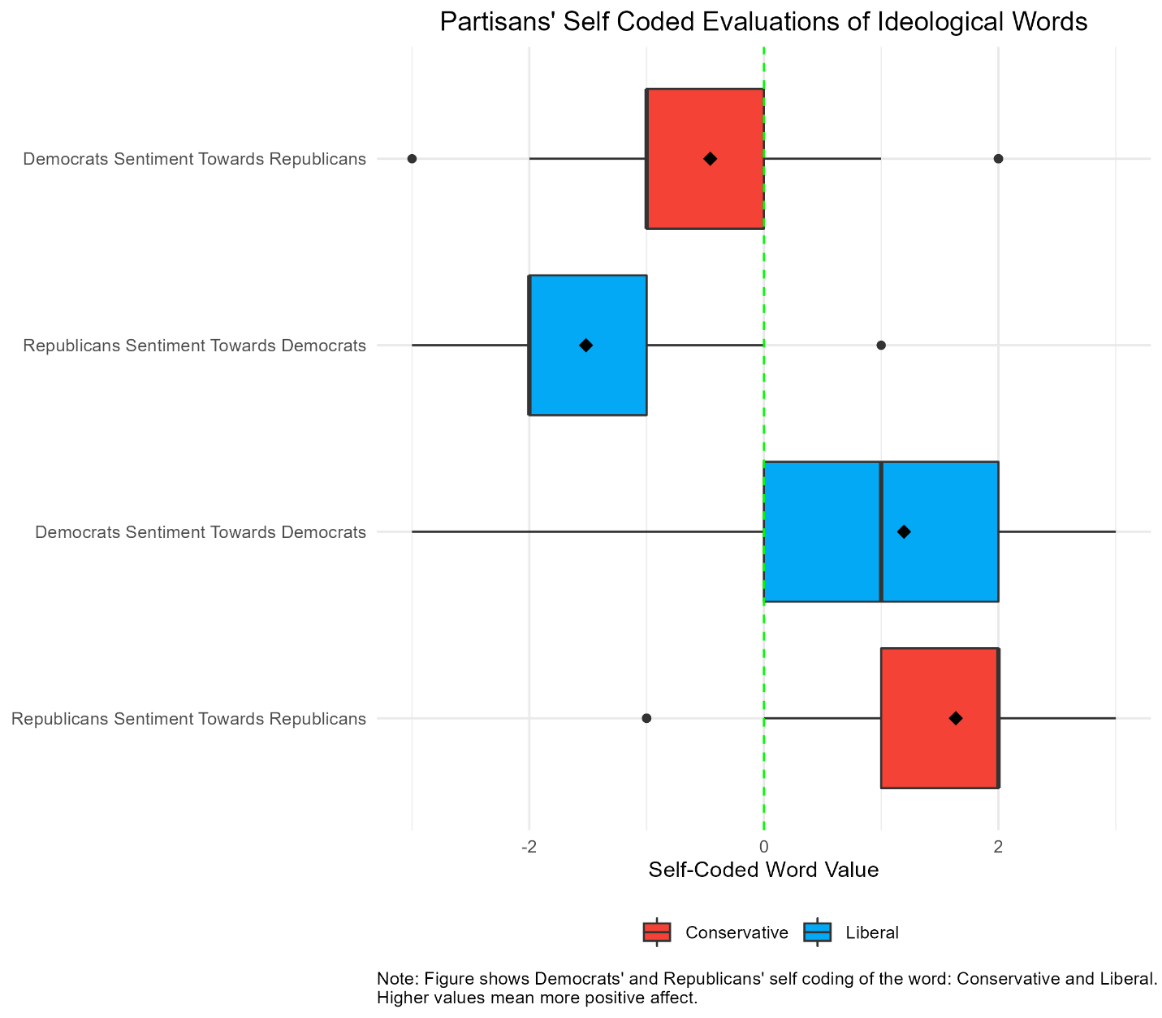


# Measures of Affective Polarization are Highly Correlated

In this section, we use our measure of affective polarization using self-coded one-word evaluations with the measures of affective polarization created from three well-established measures: thermometer scores, candidate evaluations, and lifestyle measures.

Mirroring the main text, we create each measure of affective polarization using the same formula:

$$Affective Polarization= In Party Evaluation-Out Party Evaluation$$

The formula for affective polarization uses the self-coded word and takes respondents’ in-party affect evaluation and subtracts it from a respondents’ out-party affect evaluation.

Our one-word self-coded measure of affective polarization ranges from ranging from -6 representing extreme in-party dislike to 6 representing extreme out-party dislike, with 0 indicating indifference between both parties.

The affective polarization measure using thermometer scores ranges from -100 representing extreme in-party dislike to 100 representing extreme out-party dislike, with 0 indicating indifference between both parties. The affective polarization measure using candidate evaluations ranges from -10 representing extreme dislike for the in-party’s candidate to 10 representing extreme dislike for the out-party’s candidate, with 0 indicating indifference between both candidates. The affective polarization measure using lifestyle evaluation ranges from -3 representing extreme aversion to in-party interactions to 4 representing extreme aversion to out-party interactions, with 0 indicating indifference between interacting with both parties.

Figure 2A reports that when we compare our one-word self-coded measure of affective polarization with existing measures of affective polarization, the relationship between the measures is positive and moderately strong. The correlations report a strong relationship between one-word affective polarization and thermometer affective polarization (r = 0.63), candidate affective polarization (r = 0.57), and lifestyle affective polarization (r = 0.47).

Together, these results show that our one-word measures of affective polarization are strongly related, they are not perfectly related. The conclusions we can draw form this relationship is the same as the conclusions we can draw from our comparison between affect in the main text: the residuals emphasize a different dimension of affective polarization that the one-word measures detect that the other measures do not. As a result, this makes them unique and contribution to affective polarization literature.

## Figure 2A: Scatterplot of Self-Coded One Word Evaluations Against Existing Affective Polarization Measures


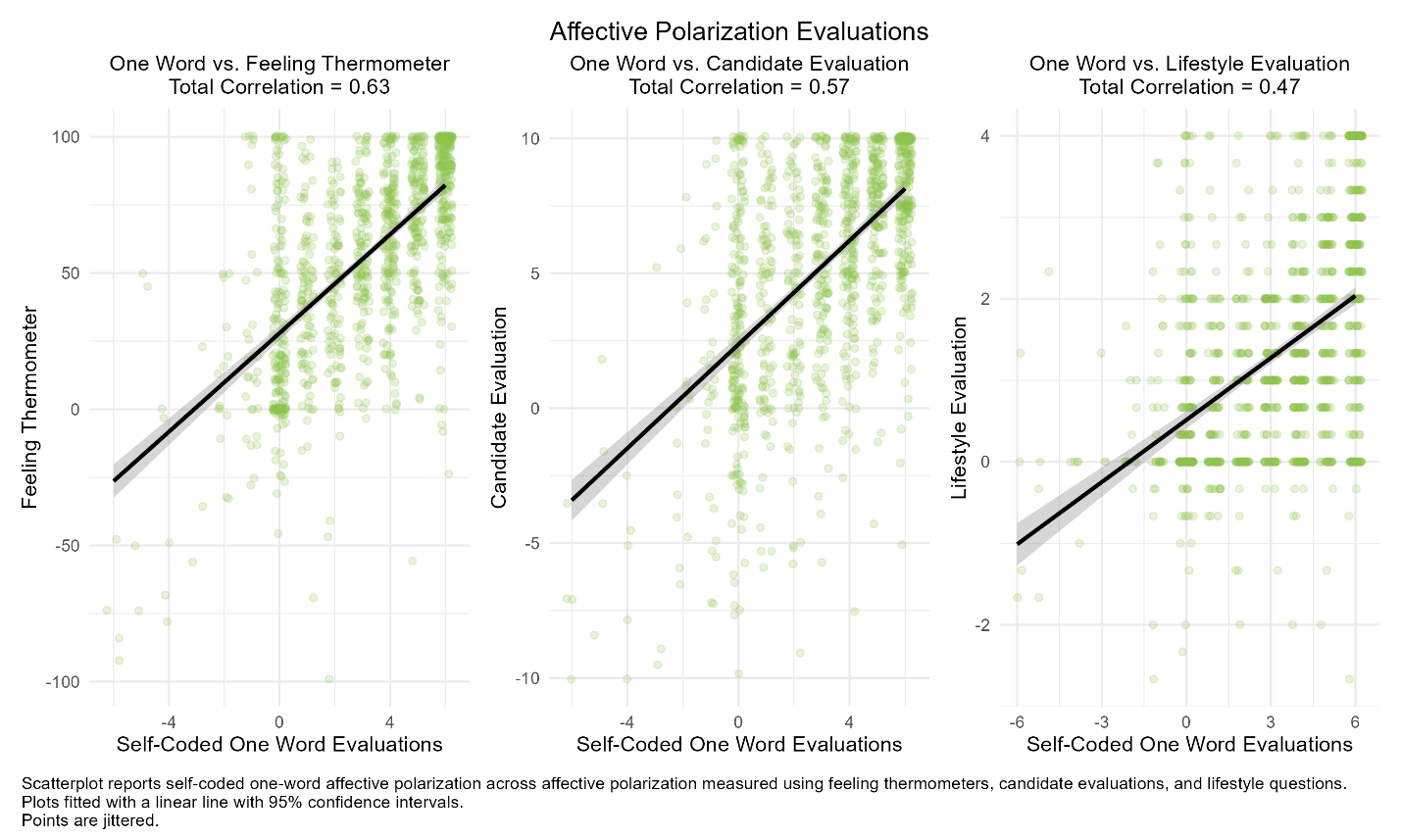


# Affective Word Dimensions Using In-Party Specification

In the main text of the paper, we examine the policy and valence dimensions using the respondents’ out-party word. Here, we keep the model specifications the same and re-run the analysis from the main text using policy and valence dimensions using the respondents’ out-party word. We find virtually no meaningful relationship between policy and valence dimension using in-party words and affective polarization. The results show that character evaluations of the in-party are not a meaningful predictor of affective polarization while policy evaluations of the in-party predict lower levels of affective polarization using candidate evaluations, social-distance measures, and more complex models of using one- word answers compared to non-policy evaluations. These results suggest that affective polarization is mostly drive by character evaluations of the out-party not character evaluations of the in-party.

## Figure 3A: Estimates for the Effect of In-Group Policy and Valence Dimension on Affective Polarization


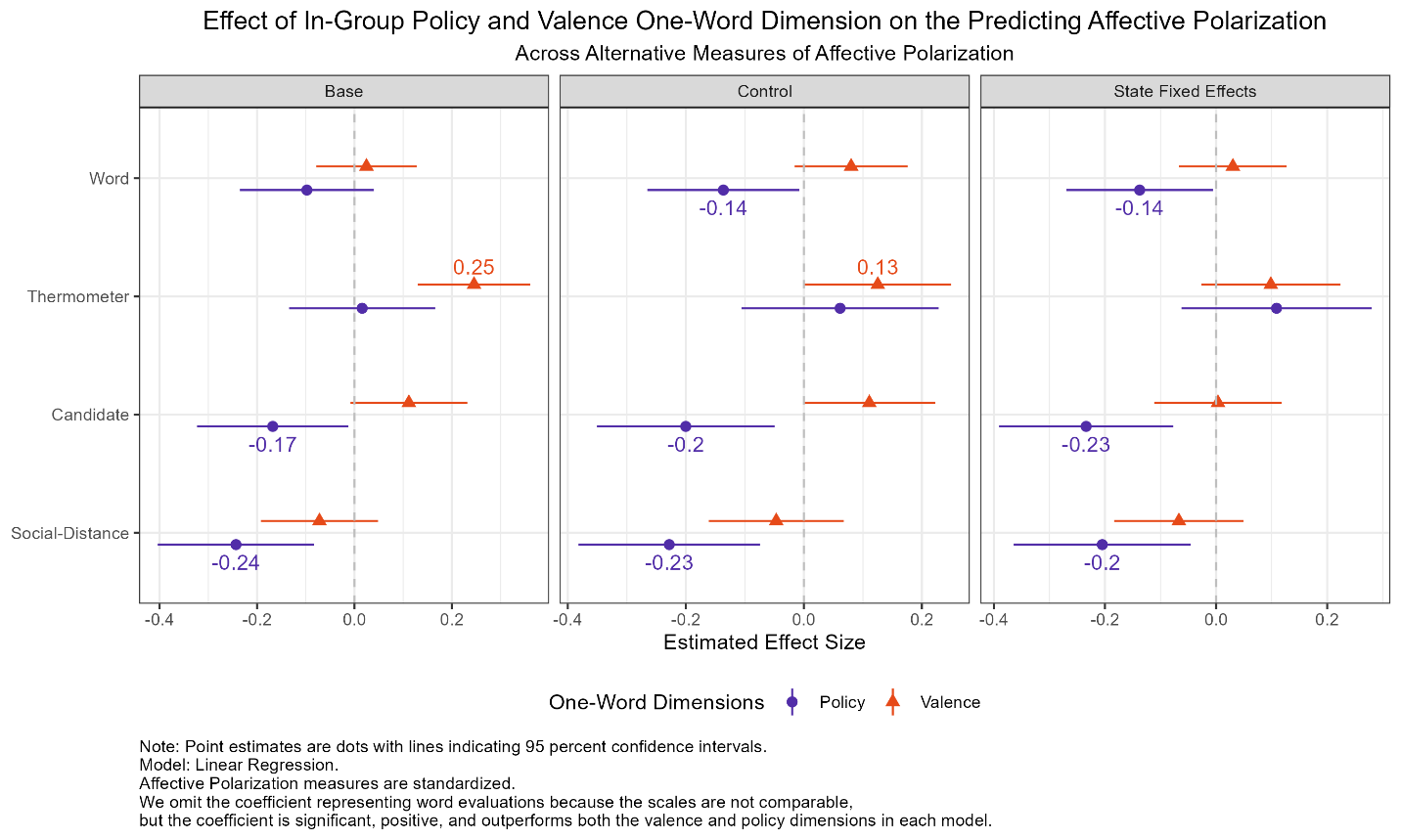


# The Benefits of Open-Ended Responses With Self-Coded Words

As opposed to closed-answer responses, which may be easier to analyze but can suffer from measurement error or internal validity issues (2,3), we rely on open ended questions. By doing so, we allow respondents to use open-ended responses to explain their state of mind unrestricted by an artificially generated scale. However, there are many pitfalls to relying on open-ended survey responses. Most consequently, is how they get coded.

First, researchers can hand code open-ended survey responses. Researchers who hand code open-ended responses face time and personnel costs associated with coding thousands of responses. Often, researchers need to create a detailed codebook of a pre-determined schema that researchers derive from a close reading of randomly selected responses (2,4,5). During the coding process, researchers might misinterpret the sentiment behind respondents’ word choice causing researcher-imposed measurement error (6). Given that research teams require multiple personnel, low inter-coder reliably might reduce confidence in the final coding (7).

Second, researchers can rely on a sentiment dictionary to automate coding, but this is often not a better option. Respondents tend to misspell words. These errors make it difficult to merge onto a sentiment dictionary. Respondents may also use slang or other words that, even if spelled correctly, might not exist in a sentiment dictionary. Additionally, respondents may have different affective evaluations of the same words. Together, these issues reduce the sample of codable words in researchers’ final datasets and reduces their statistical power in any analysis.

Finally, researchers may turn to more sophisticated methods, such as supervised machine learning, to code text (8). This method reduces time and personal costs and outperforms sentiment dictionaries (8). However, it can only be utilized by researchers with experience in text analysis and machine learning, which shuts out researchers who lack access to these tools.

Alternatively, researchers can ask respondents to code the sentiment of their own word (2). We argue that this method overcomes the challenges associated with using open-ended responses. This procedure takes the coding out of the researchers’ hands allowing respondents, the best judge of their own sentiment, to code the sentiment of their word. This method keeps the coding completely exogenous from any influence or bias researchers might have in the coding process. Self-coding has been found to be particularly effective in correcting biased coding of responses given by marginalized groups such as less wealthy respondents (Glazier, Boydstun, and Feezell 2021). As scholars note, letting survey respondents code their open-ended answers are likely our best estimate of respondents’ true opinion (2,9,10).

# Work Cited

1. Aronow PM, Kalla J, Orr L, Ternovski J. Evidence of Rising Rates of Inattentiveness on Lucid in 2020 [Internet]. SocArXiv; 2020 Sep [cited 2021 Nov 11]. Available from: https://osf.io/8sbe4

2. Glazier RA, Boydstun AE, Feezell JT. Self-coding: A method to assess semantic validity and bias when coding open-ended responses. Research & Politics. 2021 Jul 1;8(3):20531680211031752.

3. Reja U, Manfreda KL, Hlebec V, Vehovar V. Open-ended vs. Close-ended Questions in Web Questionnaires. 2003;19.

4. Baumgartner FR, Boef SD, Boydstun AE. The Decline of the Death Penalty and the Discovery of Innocence. New York: Cambridge University Press; 2008.

5. Simon A, Xenos M. Media Framing and Effective Public Deliberation. Political Communication. 2000 Oct;17(4):363–76.

6. Gibson JL, Caldeira GA. Knowing the Supreme Court? A Reconsideration of Public Ignorance of the High Court. The Journal of Politics. 2009;71(2):429–41.

7. Mikhaylov S, Laver M, Benoit K. Coder Reliability and Misclassification in Comparative Manifesto Project Codings. 66th MPSA annual national conference. 2008 May 1;

8. Barberá P, Boydstun AE, Linn S, McMahon R, Nagler J. Automated Text Classification of News Articles: A Practical Guide. Polit Anal. 2021 Jan;29(1):19–42.

9. Geer JG. What Do Open-Ended Questions Measure? The Public Opinion Quarterly. 1988;52(3):365–71.

10. Zaller J, Feldman S. A Simple Theory of the Survey Response: Answering Questions versus Revealing Preferences. American Journal of Political Science. 1992;36(3):579–616.
